# The whole person beneath the drapes: a philosophical reflection on human-centeredness in the operating room

**DOI:** 10.1186/s13010-025-00202-1

**Published:** 2025-11-05

**Authors:** Saeid Amini Rarani

**Affiliations:** Student Research Committee, Nursing and Midwifery Care Research Center, School of Nursing and Midwifery, Isfahan University of Medical Sciences, Isfahan, Islamic Republic of Iran

**Keywords:** Person-centered care, Patient dignity, Anesthesia ethics, Carl rogers, Cartesian dualism, Surgical philosophy

## Abstract

**Background:**

In the highly technical and time-pressured environment of the operating room (OR), patients may risk becoming physically present yet experientially absent and ontologically overlooked. “Experiential absence” refers to the loss of the patient’s subjectivity when their voice and awareness are silenced under anesthesia, while “ontological absence” refers to the erosion of their recognition as a person of inherent moral worth. Drapes, protocols, and clinical shorthand can unintentionally reinforce this absence. While such detachment supports surgical focus, it raises pressing ethical questions.

**Methods:**

This paper undertakes a philosophical reflection drawing on René Descartes’ *Meditations* and Carl Rogers’ humanistic psychology. Primary texts from both authors are engaged to examine how Cartesian dualism has shaped depersonalizing tendencies in biomedicine, and how Rogers’ principles of empathy, authenticity, and unconditional positive regard can reframe ethical care in surgery.

**Results:**

Three interrelated dimensions are identified. First, the paper distinguishes between necessary clinical objectivity and harmful detachment, arguing that the latter undermines ethical regard. Second, it reconceptualizes the anesthetized patient as morally present despite unconsciousness, emphasizing that vulnerability under anesthesia heightens the ethical duty of care. Third, it reframes the surgical team as a therapeutic environment, where interpersonal respect and psychological safety influence how patient dignity is upheld.

**Conclusions:**

Integrating Rogers’ philosophy into the OR does not compromise technical precision but deepens it with moral clarity. By recognizing both the experiential silence and the ontological presence of the anesthetized patient, surgical teams can align technical excellence with ethical responsibility. This perspective expands patient-centered care into a more robust person-centered ethic, positioning surgery not only as a technical intervention but also as a profoundly moral encounter.

## Introduction

In the operating room (OR), the patient is surrounded by drapes, technology, and tightly choreographed routines. Within this environment, the person beneath the surgical field can be overshadowed by anatomy, diagnosis, and procedure. A patient who has entrusted their body to the team may no longer be addressed by name or story but as “the gallbladder in Room 3” or “the fractured femur.” Such shorthand reflects more than efficiency: it illustrates a tendency to reduce patients to biological objects rather than recognize them as full moral subjects [1, 2].

This reduction is partly rooted in the philosophical legacy of René Descartes. In the *Second* and *Sixth Meditations*, Descartes famously divided the human being into a res cogitans (thinking substance) and res extensa (extended substance). This separation laid the groundwork for a biomedical model that prioritizes the repair of the body as machine, while sidelining the lived experience of the person [3]. In surgery, where patients are anesthetized and silent, this dualism becomes stark: the body remains, but the conscious self seems absent [4].

Yet absence here is not simple. Two forms must be distinguished. The anesthetized patient is experientially absent—their voice, awareness, and agency are suspended [5]. At the same time, they risk being treated as ontologically absent—as if their moral worth itself were diminished by unconsciousness. While experiential absence is an unavoidable fact of anesthesia, ontological absence is an ethical failure: it arises when the team forgets that a voiceless body remains a person of dignity [6].

Carl Rogers’ humanistic psychology offers a corrective. In works such as *On Becoming a Person* (1961) and *A Way of Being* (1980), Rogers insisted on the indivisibility of the person and the centrality of empathy, authenticity, and unconditional positive regard in any helping relationship. Although he did not write about surgery, his philosophy challenges the reduction of patients to their physiological state. For Rogers, presence is not contingent on speech or awareness; it is a moral stance toward the whole person [7, 8].

The aim of this paper is therefore twofold. First, it examines how depersonalization in the OR reflects lingering dualistic assumptions that render anesthetized patients experientially and ontologically absent. Second, it explores how Rogers’ principles can help reframe surgical care as a moral encounter, even when the patient is unconscious. In doing so, the paper argues that surgery need not be understood only as a technical intervention. It can also be a space where technical excellence is joined to ethical presence, ensuring that the person beneath the drapes is not forgotten but honored.

### Philosophical framework

#### Cartesian dualism and its surgical legacy

René Descartes, in his *Second* and *Sixth Meditations*, articulated a profound separation between mind (res cogitans) and body (res extensa). This distinction helped shape the development of modern science and medicine by allowing the body to be studied as a machine, independent of consciousness or subjective meaning. While this move advanced surgical knowledge and technical mastery, it also laid the groundwork for a culture in which patients may be treated as collections of organs and systems rather than as whole persons [9].

In the OR, this dualism is enacted most vividly under anesthesia. The patient, unable to speak or respond, is often described through clinical shorthand— “the liver transplant,” “the hip fracture in OR 2.” Such language reflects what this paper calls experiential absence (the unavoidable suspension of the patient’s voice and awareness) and, more troublingly, ontological absence (the risk that the team perceives and treats the patient as less than a moral subject). The former is a clinical reality; the latter is an ethical danger. While surgical precision requires objectivity, detachment carried too far can erode recognition of the personhood that persists beneath unconsciousness [1].

Critics have noted that Cartesian dualism does little actual explanatory work in such contexts: regardless of metaphysical views, anesthetized patients are unconscious. Yet dualism’s legacy persists not as doctrine but as habit—embedded in clinical language, organizational culture, and implicit attitudes that equate unconsciousness with diminished personhood. Here, dualism is invoked not as a metaphysical claim but as a heuristic lens, helping to understand subtle cultural patterns that influence perception of anesthetized patients. Naming this legacy clarifies how a philosophical orientation can subtly shape practical care.

#### Rogers’ humanistic corrective

Carl Rogers (1902–1987), a founding figure of humanistic psychology, rejected objectifying models of human beings and emphasized the indivisibility of the person. In *On Becoming a Person* (1961) and *A Way of Being* (1980), he described the core conditions of effective helping relationships: empathy, authenticity (congruence), and unconditional positive regard. These were not merely therapeutic techniques but attitudes toward the other’s inherent dignity and worth [7, 8].

Although Rogers wrote in the context of psychotherapy, his philosophy is not limited to conscious dialogue. Presence, for Rogers, is a moral stance: to regard the other as fully human regardless of their capacity to respond. This makes his thought strikingly relevant to surgical contexts [10]. Even when a patient is anesthetized, their ontological presence—their status as a person—remains intact. For Rogers, unconsciousness does not erase humanity; it intensifies the ethical obligation of the caregiver to uphold dignity and respect.

Bringing Rogers into dialogue with surgery provides a corrective to the residual dualism of modern medicine. Where Cartesian thinking risks fragmenting the patient into body and absent mind, Rogers reunites them as a whole person. His emphasis on relational presence offers a framework for viewing the OR not merely as a technical site of intervention but as a moral space, where every gesture, word, and relational dynamic carries ethical significance.

### The mask of objectivity: clinical detachment and the loss of personhood

Surgical practice requires precision, discipline, and a degree of emotional control. Objectivity in this context is indispensable: without it, technical accuracy and patient safety would be compromised. Yet when objectivity slides into detachment, and emotional neutrality hardens into impersonality, something essential is lost—the recognition of the patient as a person of dignity [11]. 

This loss is often visible in the language of the OR. Patients may be referred to by diagnosis or anatomy: “the appendectomy in Room 5,” “the fractured hip in OR 2.” While such shorthand supports efficiency and reduces ambiguity, it also normalizes a subtle depersonalization. The risk is not overt cruelty but a gradual erosion of relational and ethical awareness. Repeated often enough, such language can shape how clinicians perceive the patient—less as a subject of care, more as a clinical case [1].

Carl Rogers’ principle of unconditional positive regard challenges this tendency. For Rogers, respect for the other does not depend on their alertness, productivity, or communicative ability; it arises from their inherent worth as persons. Even under anesthesia, patients remain morally present. To recognize them as such requires not only technical vigilance but also ethical presence—an intentional awareness that the body under the drapes belongs to a human being with history, values, and relationships [10, 12].

Clinical detachment can feel protective in the high-pressure atmosphere of the OR. It creates emotional distance and helps staff manage stress. But there is a moral cost: indifference. When clinicians shield themselves by reducing the patient to a case, they risk neglecting the invisible labor of dignity. Small acts—a respectful silence, careful handling of the body, avoiding casual jokes at the patient’s expense—can affirm personhood even when the patient cannot perceive them [13].

Here, professionalism should not be equated with the suppression of emotion, but with the integration of moral consciousness into technical practice. As Rogers insisted, presence is not passive; it is active, deliberate, and courageous. In the OR, maintaining presence means holding in view the whole person, even when unseen, unheard, and unresponsive [14].

To “remember the person behind the procedure” is therefore not a distraction from technical excellence but a way of refocusing it. Aligning surgical skill with ethical clarity ensures that the discipline of detachment serves safety without eroding humanity [15].

### Human presence under anesthesia: trust, vulnerability, and moral attention

Anesthesia introduces one of the most profound ethical challenges in medicine. When consciousness is suspended, the patient is experientially absent—unable to speak, perceive, or advocate for themselves. Yet this does not mean they are ontologically absent. Their moral presence endures, and with it the obligations of respect and dignity [16].

Under anesthesia, vulnerability reaches its peak. The anesthetized patient cannot signal discomfort, challenge decisions, or object to violations of privacy. They must entrust their body, identity, and safety entirely to others. This creates what can be described as a sacred asymmetry of trust. The silence of the anesthetized is not emptiness but entrustment. It calls for heightened ethical presence on the part of the surgical team [17].

Here Carl Rogers’ philosophy offers a critical insight. Rogers argued that presence is not contingent on dialogue or awareness; it is an intentional stance of respect toward the other. In A Way of Being (1980), he described presence as a quality of openness and attentiveness that affirms the other’s worth without condition [8]. Transposed to the surgical context, this means that even when the patient cannot respond, the team’s way of speaking, touching, and behaving communicates whether the patient is treated as a body or as a person [17].

Empirical research supports this moral claim. Studies show that small acts—covering the patient respectfully with drapes, avoiding casual or objectifying language, maintaining a calm and respectful atmosphere—contribute not only to patient dignity but also to staff professionalism and team cohesion. Such gestures may leave no trace in the patient’s conscious memory, but they shape the moral character of caregivers and the ethical climate of the OR [18].

The danger in anesthesia is not deliberate harm but unnoticed indifference. When the patient cannot hear or respond, it becomes easier for clinicians to disengage ethically, to let objectivity blur into impersonality. Yet it is precisely in this moment of silence that moral attention matters most [19].

To regard the anesthetized patient as a full moral subject is therefore not sentimental idealism but ethical realism. Vulnerability without voice intensifies, rather than diminishes, the obligation of care. In this light, anesthesia is not the erasure of the person but the moment when the ethical weight of personhood is most clearly entrusted to the surgical team.

### The team as therapeutic environment: psychological safety and ethical climate in the OR

Carl Rogers emphasized that personal growth and healing depend not only on individual qualities but also on the relational climate. In psychotherapy, this meant creating an environment characterized by empathy, congruence, and unconditional positive regard [7]. In the operating room, a comparable principle applies: the OR team itself constitutes a therapeutic environment, shaping how the patient is regarded and treated.

The anesthetized patient is embedded within this micro-culture. Although unconscious, their experience of care is mediated through the attitudes, language, and behaviors of those around them. A team marked by mutual respect, psychological safety, and open communication is more likely to uphold patient dignity. Conversely, when cynicism, hierarchy, or emotional suppression dominate, depersonalization can become normalized [20].

Recent literature underscores the practical relevance of this point. Studies demonstrate that psychological safety within surgical teams improves both technical outcomes and ethical vigilance. When nurses, anesthetists, and junior surgeons feel empowered to speak up, they are more likely to challenge depersonalizing language, advocate for respectful practice, and attend to small but meaningful acts of care. Ethical attention, in this sense, is not only an individual responsibility but also a systemic property of the team environment [21, 22].

Rogers’ vision of a therapeutic climate offers concrete guidance. In the OR, this might mean that a senior surgeon invites and respects contributions from colleagues, fostering mutual regard. It may involve a circulating nurse ensuring that the patient’s body is positioned and covered respectfully, even though the patient is unconscious. These seemingly minor actions signal a culture where presence and dignity matter as much as precision and speed [23]. By consciously integrating these small but deliberate practices, the OR team operationalizes Rogers’ humanistic principles, ensuring that technical excellence is aligned with ethical attentiveness.

Importantly, such a climate cannot be sustained by policy alone. It requires daily ethical labor: reflective self-awareness, deliberate team practices, and resistance to depersonalizing norms. Recognizing the ethical labor of nurses and technicians—often the most consistent advocates for patient dignity—reinforces the idea that the surgical field is not only a technical zone but also a moral space [24].

In this light, the OR team functions as a moral community. Its collective posture determines whether the anesthetized patient is seen merely as an object of intervention or as a person entrusted to shared responsibility. When the team embodies Rogers’ principles, the surgical encounter becomes not only a technical achievement but also an ethical one.

## Conclusion and implications

### Reclaiming the human in surgical care

The operating room is often imagined as a domain of science and control—a space where bodies are repaired with technical precision and emotional detachment. Yet beneath the sterile drapes lies not merely a biological object, but a person: a being with history, values, vulnerabilities, and dignity. This paper has argued that even when a patient is unconscious, their experiential absence must not be mistaken for ontological absence. The patient’s moral presence endures, calling for ethical recognition from the surgical team.

Drawing on René Descartes and Carl Rogers, I have highlighted two philosophical orientations with contrasting implications. The Cartesian legacy, while enabling biomedical progress, risks fragmenting the person into body and mind, fostering depersonalization when consciousness is suspended. Rogers’ humanistic psychology offers a corrective: his principles of empathy, authenticity, and unconditional positive regard affirm the patient as a whole person, even in silence [7, 25].

Three ethical insights emerged from this reflection. First, clinical objectivity is essential, but detachment that erases personhood undermines care. Second, anesthesia does not diminish moral presence but amplifies the duty of respect and attentiveness. Third, the OR team itself functions as a therapeutic environment, where psychological safety and interpersonal respect shape the culture of care.

These insights have practical implications. They invite surgical teams to extend patient-centered care into a more robust person-centered ethic, even in the context of a “one-shot” surgical encounter. This does not weaken technical focus; rather, it enriches it by aligning skill with moral clarity. Concrete practices—such as respectful language, attentive handling of the body, fostering psychological safety, and acknowledging the patient’s vulnerability—can sustain this alignment.

Education and training should also be reimagined. Beyond compliance with ethical guidelines, surgical teams require formation in reflective practice, moral imagination, and team-based ethical awareness. Supporting nurses, anesthetists, and junior staff in voicing ethical concerns is vital for sustaining a culture where dignity is not incidental but central.

Ultimately, honoring the person beneath the drapes is not an optional ideal but a professional imperative. By situating Rogers’ humanism within the high-stakes context of anesthesia, this paper extends person-centered ethics into one of the most depersonalized spaces of modern medicine. Surgery is unavoidably technical, but it is also irreducibly moral. To integrate Rogers’ humanism into the OR is to affirm that healing is not only a matter of hands and instruments, but of presence, respect, and relationship. In doing so, surgical teams ensure that technical excellence and ethical responsibility are not competing demands, but mutually reinforcing dimensions of care.

## Data Availability

No datasets were generated or analysed during the current study.
